# Angioimmunoblastic T-Cell Lymphoma with Exuberant CD30-Positive Follicular Dendritic Cell Proliferation in a SARS-CoV-2 Patient: The Role of Mutational Analysis to Exclude an Associated Follicular Dendritic Cell Sarcoma

**DOI:** 10.3390/ijms23169349

**Published:** 2022-08-19

**Authors:** Evelina Rogges, Sabrina Pelliccia, Gianluca Lopez, Sabina Barresi, Agostino Tafuri, Rita Alaggio, Arianna Di Napoli

**Affiliations:** 1 Pathology Unit, Department of Clinical and Molecular Medicine, Sant’Andrea University Hospital, Sapienza University of Rome, 00189 Rome, Italy; 2 Hematology Unit, Department of Clinical and Molecular Medicine, Sant’Andrea University Hospital, Sapienza University of Rome, 00189 Rome, Italy; 3 Pathology Unit, Department of Laboratories, Bambino Gesù Children’s Hospital, Istituto di Ricerca a Carattere Scientifico (IRCCS), 00165 Rome, Italy; 4 Department of Medico-Surgical Sciences and Biotechnologies, Sapienza University of Rome, 00185 Rome, Italy

**Keywords:** AITL, follicular dendritic cell proliferation, CD30 expression, SARS-CoV-2 infection

## Abstract

Follicular dendritic cell (FDC) proliferation in angioimmunoblastic T-cell lymphoma (AITL) is still not well defined, challenging the accurate differential diagnosis between the AITL with expanded follicular dendritic cell meshwork and the combined AITL and follicular dendritic cell sarcoma (FDCS). Herein, we reported the case of a 58-year-old male with coexisting SARS-CoV-2 infection and AITL with an exuberant CD30-positive FDC proliferation, in which genetic analysis identified mutations of genes commonly involved in AITL but not in FDC sarcoma (i.e., RHOA, TET2, DNMT3A, and IDH2), thus supporting the reactive nature of the CD30-positive FDC expansion.

## 1. Introduction

Angioimmunoblastic T-cell lymphoma (AITL) [1], recently renamed as nodal follicular T helper (TFH) cell lymphoma, angioimmunoblastic-type (nTFHL-AI) in the upcoming World Health Organization classification of hematolymphoid tumors [2], is a node-based peripheral T-cell lymphoma of middle-aged and elderly individuals characterized by generalized lymphadenopathy, organomegaly, fever, weight loss, pruritus, skin rash, anemia, and polyclonal hypergammaglobulinemia. Histologically, the normal architecture of the lymph node is partially or totally effaced by a polymorphous cellular infiltrate composed of atypical T cells accompanied by an inflammatory background of reactive small lymphocytes, immunoblasts, plasma cells, mast cells, eosinophils, and histiocytes. The neoplastic T-cell infiltrate is mostly represented by small- to medium-sized CD4-positive T cells with clear cytoplasm and mild nuclear atypia, frequent loss of CD7, and expression of at least two of the TFH markers CD10, BCL6, PD1, ICOS, and CXCL13. Three morphological patterns are recognized: in Pattern 1, the neoplastic T-cell infiltrate surrounds hyperplastic follicles; in Pattern 2, the paracortex is expanded, and the follicles show regressive changes; in Pattern 3, the extensive infiltration of the neoplastic T cells associated with the prominent proliferation of high endothelial venules (HEVs) and the expansion of follicular dendritic cells (FDCs) prevents the presence of follicles [1]. However, the extent of the FDC proliferation and the border with FDC sarcoma remain unclear.

FDC sarcoma (FDCS) is an uncommon malignant neoplasm occurring in lymph nodes and extranodal sites originating from germinal centers with stroma-derived perivascular progenitors expressing the FDC markers CD21, CD23, and CD35 [3]. Morphologically, spindled, ovoid, or epithelioid neoplastic cells with elongated nuclei and vesicular chromatin are arranged in whorls, fascicles, syncytial sheets, or nodules with a storiform pattern. FDCS has been described in association with hyaline-vascular Castleman disease [4], follicular lymphoma [5], and chronic lymphocytic leukemia/small lymphocytic lymphoma [6]. Only two case reports questioned about the synchronous occurrence of FDCS in patients with T-cell lymphomas in the absence of extensive molecular information [7,8]. AITL, in fact, are frequently characterized by peculiar genetic changes such as mutations in epigenetic modifiers (TET2, DNMT3A, IDH2) and in T-cell receptor (TCR) signaling molecules (RHOA, VAV1, PLCG1, FYN, CD28) [9]. In contrast, alterations of genes involved in the NFkB pathway (BIRC3, NFKBIA, TRAF3, SOCS3, CYLD, and TNFAIP3) and of tumor suppressor genes (CDKN2A, RB1, and TP53) recur in FDCS [10].

CD30 is a transmembrane cytokine receptor belonging to the tumor necrosis factor (TNF) receptor that can be expressed by T or B cells in different benign and neoplastic conditions [11]. In FDCS. scattered CD30-positive spindle cells have been described in rare cases [12,13,14,15]; in contrast, CD30 is invariably negative in non-neoplastic FDC.

We describe the case of an exuberant expansion of CD30-positive FDC in a patient with AITL and symptomatic SARS-CoV-2 infection, in which the genetic analysis identified the presence of mutations only in genes usually associated with AITL and not with FDCS.

## 2. Results

### 2.1. Clinical Data

On 11 March 2020, at the beginning of the COVID-19 pandemia in Italy, a 58-year-old patient presented at the emergency room of Sant’Andrea Hospital of Rome with fever and dyspnea. Recent clinical history revealed weight loss of seven kilograms in the previous three months, but no other symptoms. Blood work showed marked lymphopenia and negativity for toxoplasma, cytomegalovirus, Epstein–Barr virus, and human immunodeficiency virus infection. Because of the patient’s compromised respiratory function, a total-body computed tomography (TBCT) scan was performed, which showed severe bilateral cervical, axillary, and mediastinal lymphadenopathy (maximum diameter 4 cm), hepatomegaly, bilateral pleural effusion, and a displaced peripheral segmental and round ground-glass opacity in both lungs with inter- and intralobular septal interstitial thickening (“crazy paving” pattern). Although these findings were highly suggestive of COVID-19 pneumonia, the molecular test for the research of SARS-CoV-2 virus RNA (nasopharyngeal swab) resulted negative. Therefore, the diagnostic workup proceeded with the biopsy of two cervical lymph nodes. Three days later, the repeated test resulted in a positive value, and the patient was treated with azithromycin, corticosteroids, heparin, hydroxychloroquine, and Darunavir–Cobicistat according to treatment strategies for SARS-CoV-2-infected patients in that period. Due to the persistence of the symptoms and the high blood levels of IL-6, tocilizumab was also administered. A subsequent positron emission tomography scan (PET) showed a diffuse radioconcentration in the lungs and pathological uptake in laterocervical and ilo-mediastinal lymph nodes bilaterally and also in the left retroangolomandibular and supraclavicular lymph nodes (SUV max 8.52).

### 2.2. Histology

**Figure 1:**
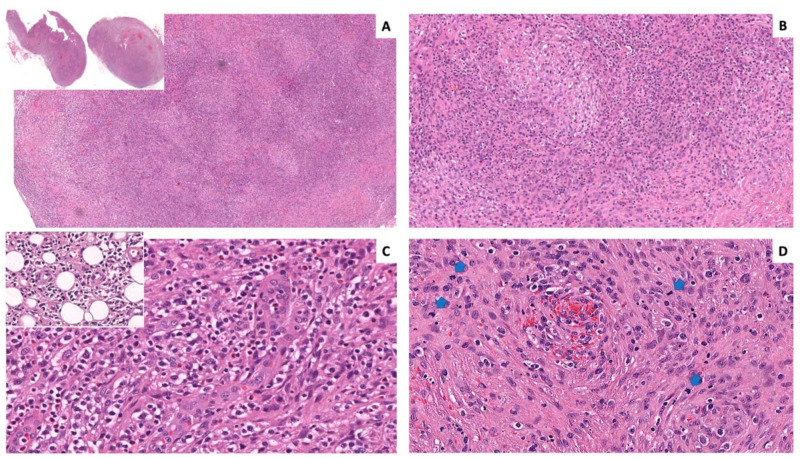
Histology of the lymph nodes. Pale and dark areas were evident at even low magnification ((**A**) hematoxylin and eosin (H&E) original magnification (o.m.) ×16, insert o.m. ×5; (**B**) H&E o.m. ×66). The dark areas were populated by atypical lymphocytes with clear cytoplasm clustered around HEVs and infiltrating the perinodal adipose tissue (insert). Admixed small lymphocytes, histiocytes, and eosinophils were also present ((**C**) H&E o.m. ×200; insert ×200). The pale areas were composed of whorls of proliferating spindled and ovoid cells arranged around blood capillaries ((**D**) H&E o.m. ×200; blue arrows indicate mitotic figures).

Histological examination of the excised lymph nodes revealed complete effacement of the architecture with a pseudonodular appearance due to the alternation of dark and pale areas (Figure 1A,B). The darkest zones were composed of medium-sized round lymphocytes, with abundant eosinophilic to clear cytoplasm that frequently formed small clusters and extended through the lymph node capsule into the surrounding adipose tissue (Figure 1C). In the palest pink areas, there was a diffuse network of spindled to ovoid cells with moderate amounts of eosinophilic cytoplasm, formed a dense pink band at the periphery of the lymph node with focal nodularity (Figure 1B,D). The cells displayed elongated or oval nuclei with low-to-mild atypia and evident mitotic activity. Both cell populations were arranged around blood vessels. No germinal centers were noted.

**Figure 2:**
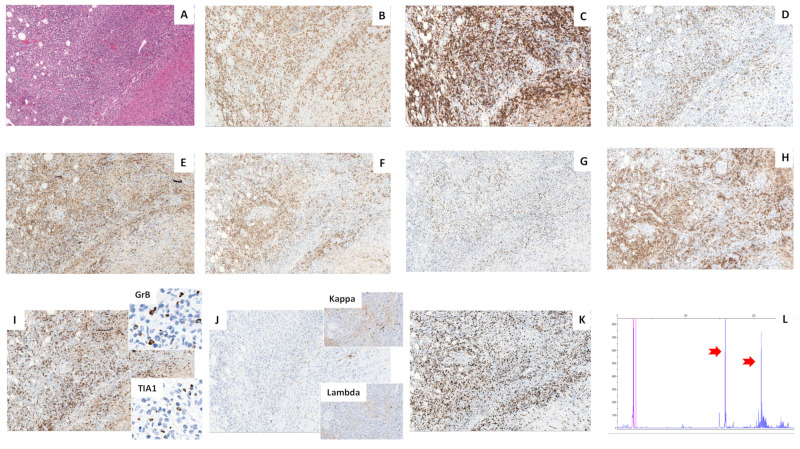
Immunohistochemistry and clonality analysis of the T-cell component. The atypical lymphocytes ((**A**) H&E, o.m. ×66) express CD3 and CD5 and showed partial loss of CD7 ((**B**–**D**) o.m. ×66). The CD4+, CD10+, BCL6+, PD1+ ((**E**–**H**) o.m. ×66) cells outnumber the CD8+, TIA1+, GrB+ T cells ((**I**) o.m. ×66; inserts o.m. ×200). The B-cell component was constituted by occasional small CD20+ cells and rare polytypic plasma cells ((**J**) o.m. ×66; inserts o.m. ×66). Staining for Ki67 showed a high proliferation index (70*–*80%) ((**K**) o.m. ×66). The molecular gene scan analysis showed two high peaks (red arrows) suggestive of a monoclonal biallelic*/*biclonal rearrangement of the TRG gene (**L**).

**Figure 3:**
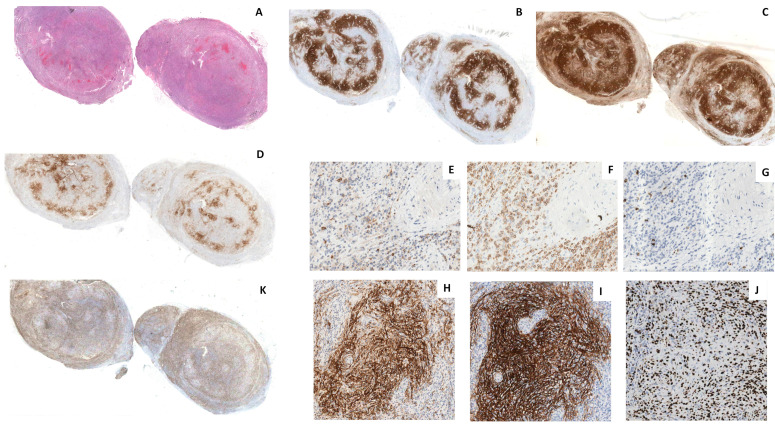
Immunohistochemistry of the FDC component. Pale fascicles and nodules ((**A**) H&E) of FDCs were highlighted by both CD21 (**B**) and CD23 (**C**) stainings (o.m. ×5). CD30 ((**D**) o.m. ×5) stained sparse medium/large lymphocytes ((**E**) CD30 o.m. ×200), mainly of the T-cell lineage ((**F**) CD3, o.m. ×200) as shown by the rarity of the B cells ((**G**) CD79a o.m. ×200), and numerous FDC ((**H**) CD30 o.m. ×100; ((**I)** CD21 o.m. ×100). The Ki-67 index ((**K**) o.m. ×5) in the FDC areas was about 40%; ((**J**) o.m. ×100).

The immunohistochemical evaluation highlighted the presence of two predominant populations. The atypical lymphocytes had a T immunophenotype (CD3+, CD5+, CD4+) with partial loss of CD7 and expression of the TFH markers CD10, BCL6, and PD1 (Figure 2A–H), whereas the pale areas were composed of CD23+ and CD21+ FDCs (Figure 3A–C). Intermixed to these two components, there was a polymorphous inflammatory background containing variable amounts of reactive small cytotoxic -cells (CD8+, TIA1+, Granzyme B+) (Figure 2I), scattered CD20+ and CD79a+ B lymphocytes, rare CD138+ polytypic plasma cells (Figure 2J), histiocytes, and eosinophils. A high proliferation index was demonstrated by staining for Ki67 (Figure 2K). Surprisingly, numerous FDC cells and some T lymphocytes showed strong membrane expression of CD30 (Figure 3D–I), but negativity for CD15, EBER, and LMP1. The bone marrow trephine biopsy revealed the presence of only scattered, small CD4+, and CD8+ T lymphocytes. A diagnosis of AITL with pattern III morphology was suspected.

### 2.3. Molecular Analyses

To confirm the diagnosis of AITL, clonality studies were performed using a gene scan polymerase chain reaction approach (TCRg rearrangements molecular analysis kit, MasterDiagnostica, Granada, Spain, and IdentiClone IGH geneclonality, Invivoscribe Inc., San Diego, CA, USA). The analyses showed a monoclonal biallelic/biclonal rearrangement of the T-cell receptor gamma gene (TRG) (Figure 2L) and a polyclonal rearrangement of the immunoglobulin heavy-chain gene (IGH) consistent with T-cell lymphom a.

Due to the extensive CD30+ FDC proliferation, in order to exclude an FDCS, we investigated somatic mutations by performing a targeted next-generation sequencing analysis interrogating 500 cancer-associated genes (Illumina TruSightTM Oncology 500 panel on a Nextseq550 platform). Due to the adequate content of the FDC (>20% of cellularity) and their admixture with the T cells, microdissection was not performed, and the DNA for the analysis was extracted from the whole tissue section. The median exon coverage was 1332. The data analysis, conducted using the PierianDx software, identified pathogenic variants in RHOA, DNMT3A, TET2, and IDH2, and variants with unknown significance in NSD1, PIK3C2G,. DICER1, and CDK12 (Table 1). No copy number alterations were found in the investigated genes, whereas the tumor mutational burden was 3.1.

These results showed mutations in genes (i.e., RHOA, DNMT3A, TET2, and IDH2) associated with AITL, but not those known to be involved in FDCS. Indeed, among the variants with unknown significance, only the PIK3C2G p.A724G has been reported in colon cancer. Moreover, these variations showed a high variant allele frequency (VAF) suggesting their possible germline origin.

### 2.4. Treatment and Outcome

On 26 June, after a negative COVID test, the patient was discharged from the infectious disease department, but he was hospitalized again in August because of Klebsiella KPC lung infection. Once this infective complication was resolved, the patient underwent chemotherapy, based on a CHOEP regimen (cyclophosphamide, doxorubicin, vincristine, etoposide, and prednisone), achieving complete remission. Later on, he was consolidated by high-dose therapy, followed by autologous stem cell transplantation. To date, the patient remains in complete remission and is in good general condition.

## 3. Discussion

Angioimmunoblastic T-cell lymphoma is characterized by progressive proliferation of HEV and FDC, likely sustained by the cytokines, in particular CXCL13, produced by the neoplastic T cells. Indeed, in AITL with Pattern 1 morphology, the hyperplastic follicles show subtle or no expansion of FDCs, whereas in Pattern 3, germinal centers are absent, and there is prominent FDC expansion abutting HEV [17,18]. However, in occasional cases, the FDC expansion may be so prominent to raise concern for a neoplastic FDC lesion (2, 3). Following proliferation, FDCs may acquire nuclear atypical changes, epithelioid or Reed–Sternberg-like features, and increased mitoses. These characteristics may be evidence of a hyperplasia–dysplasia–neoplasia progression of FDC proliferation, as it has been proposed in cases of hyaline-vascular Castleman disease (HVCD) with concurrent FDCS development [18,19]. In particular, in HVCD, the interfollicular overgrowth of atypical FDC may suggest evolution to a frank sarcoma, which is characterized by a nodular mass with expansive growth and dense cellularity with a predominant storiform pattern [20,21]. In our case, although the FDC proliferation showed remarkable mitotic activity, a frank cytological atypia was lacking. Moreover, in spite of evidence of small nodular aggregates and fascicles of FDC, there was no evidence of a mass forming lesion, and the FDC still appeared to be intermingled with the neoplastic T cells. In addition, the mutational profile revealed alterations of TET2, DNMT3A, IDH2, and RHOA, which were typical of AITL, but not of FDCS. It may be argued that TET2 and DNMT3A mutations are not necessarily associated with malignancy, nor lineage specific, being observed in several lineages of non-neoplastic cells, including B and T cells (so-called “clonal hematopoiesis”) [22]. Nonetheless, they have not been reported in FDCS, in keeping with the non-hematopoietic but stromal origin of FDC.

What was unexpected in the case described here was the wide CD30 expression, which has been mainly associated with B blasts or neoplastic T lymphocytes in AITL (1) and reported only occasionally in FDCS cells [12,13,14,15]. Liu et al. described the case of an FDCS initially misdiagnosed as anaplastic large cell lymphoma due to the CD30 expression by nests of large and pleomorphic tumor cells [15]. In our patient, CD30 was expressed by both the neoplastic T cells and the expanded FDC. We do not have a valid explanation for this finding. However, since CD30 can also be induced in normal T and B cells during viral infections [11], we might speculate that in our patient, characterized by a dysregulated immune system due to the AITL, the SARS-Cov-2 infection could have abnormally stimulated the FDC inducing both proliferation and CD30 expression. In keeping with this hypothesis is the finding that COVID patients have high CXCL13 blood levels [23], which is known to be produced also in AITL and crucial in stimulating FDC [24].

In conclusion, we illustrated here the first case of a patient with symptomatic SARS-CoV2 infection and concomitant AITL showing an unusual expansion of CD30-positive FDC suggested to be non-neoplastic by the combination of histological and molecular analyses. Our report underscores the importance of a thoughtful consideration of possible atypical histopathological and/or immunophenotypical features in the diagnosis of lymphomas during SARS-CoV2 infection.

## Figures and Tables

**Table 1:** Summary of the results for the targeted NGS screening with TruSight Oncology 500 assay and PierianDx software analysis.

Gene	Reference	Chr	cDNA	Consequence	AAChange	COSMIC/dbSNP *	TIER **	VAF ***
*RHOA*	NM_001664.2	3	c.50G > T	Missense	p.G17V	COSV69041529/NA	I	14%
*DNMT3A*	NM_022552.4	2	c.1015-2A > G	Splice site	p.?	rs920946076	II	27.9%
*TET2*	NM_001127208.2	4	c.5471delG	Frameshift	p.G1824Vfs*9	NA	II	30.1%
*TET2*	NM_001127208.2	4	c.1859dupA	Insertion—Frameshift	p.Y620*	COSV54426384	II	24.4%
*IDH2*	NM_002168.2	15	c.516G > C	Missense	p.R172S	COSV57468772/rs1057519736	II	16.3%
*NSD1*	NM_022455.4	5	c.4210C > T	Missense	p.R1404C	NA	III	20.3%
*PIK3C2G*	NM_004570.4	12	c.2171C > G	Missense	p.A724G	COSV5683687/rs189828472	III	50.6%
*DICER1*	NM_177438.2	14	c.3631G > A	Missense	p.V1211M	rs764470378	III	47.7%
*CDK12*	NM_016507.2	17	c.4151G > T	Missense	p.G1384V	NA	III	48.2%

* COSMIC: Catalogue of Somatic Mutations in Cancer; dbSNP: the NCBI database of genetic variation. ** Tier I, variants with strong clinical significance; tier II, variants with potential clinical significance; tier III, variants with unknown clinical significance; and tier IV, variants that are benign or likely benign [16]. *** Allele variant frequency.

## Data Availability

Not applicable.
